# Association of *FTO* Polymorphisms with Obesity and Obesity-Related Outcomes in Portuguese Children

**DOI:** 10.1371/journal.pone.0054370

**Published:** 2013-01-14

**Authors:** David Albuquerque, Clévio Nóbrega, Licínio Manco

**Affiliations:** 1 Research Centre for Anthropology and Health (CIAS), Department of Life Sciences, University of Coimbra, Coimbra, Portugal; 2 Center for Neurosciences and Cell Biology, University of Coimbra, Coimbra, Portugal; University of Santiago de Compostela School of Medicine – CIMUS, Spain

## Abstract

**Background:**

Several studies have reported an association between single nucleotide polymorphisms in the first intron of the *FTO* gene and body mass index (BMI) or obesity. However, this association has not yet been studied among the Portuguese population. This study aims to assess the association of three *FTO* polymorphisms (rs1861868, rs1421085 and rs9939609) with obesity-related outcomes in a sample of Portuguese children.

**Methods:**

We examined a total of 730 children, 256 normal-weight (55.9% girls), 320 overweight (45.3% girls) and 154 obese (53.2% girls), aging from 6 to 12-years-old, recruited randomly from public schools in the central region of Portugal. DNA samples were genotyped for the three polymorphisms by allelic discrimination TaqMan assay. Association of the *FTO* polymorphisms with several anthropometric traits was investigated. Additionally, we tested association with the risk of obesity using overweight and obese *vs*. normal-weight children.

**Results:**

We found significant associations of rs9939609 and rs1421085 polymorphisms with weight, BMI, BMI Z-score, waist circumference and hip circumference, even after age and gender adjustment (*p*<0.05 in all traits). For rs1861868 polymorphism, marginally significant associations were obtained with weight (*p* = 0.081) and BMI (*p* = 0.096) after adjustment for age and gender. In case-control studies, both rs9939609 and rs1421085 polymorphisms were significantly associated with obesity (OR 1.97; 95% CI, 1.08–3.59; *p* = 0.026; OR 2.11; 95% CI, 1.17–3.81; *p* = 0.013, respectively) but not with overweight (*p*>0.05). Haplotype analyses identified two combinations (ACA and GCA) associated with a higher risk of obesity (OR 1.53; 95% CI, 1.06–2.22; *p* = 0.023; OR 1.73; 95% CI, 1.06–2.87; *p* = 0.030, respectively).

**Conclusions:**

This study provides the first evidence for the association of *FTO* polymorphisms with anthropometric traits and risk of obesity in Portuguese children.

## Introduction

Overweight and obesity are a major health issue associated with risk factors for the development of hypertension, type 2 diabetes and cardiovascular diseases [1]. This complex phenotype results from the interaction of environmental and multiple genetic factors influencing body mass index (BMI), with heritability estimated at 40–70% [2].

The advent of Genome Wide Association Studies (GWAS) emerged as a powerful approach to identify genetic variants associated with common diseases [3]. Until now, GWAS deliver the identification of at least 52 genetic loci robustly associated with obesity [4]. In 2007, a strong association was detected between common single nucleotide polymorphisms (SNPs) in the first intron of the fat mass and obesity-associated gene (*FTO*), on the chromosome 16q12.2, and risk of obesity [5], [6]. Of those SNPs, the rs9939609 is one of the most extensively studied, explaining about 1% of BMI heritability [5]. Each rs9939609-A allele in this gene increases body weight by 1.5 kg in adult, with similar effects observed in children and adolescents [5]. Subsequently, several other studies have consistently confirmed the association of a cluster of SNPs within the first intron of the *FTO* gene with obesity-related traits in several European [5]–[10], Asian [11]–[15] and African [16], [17] populations.

Knowledge of the genetic risk factors associated with common childhood obesity, can be helpful to design prevention strategies. Although in the Portuguese population several studies were made concerning the prevalence of overweight and obesity [18], [19], until now, no studies reporting the association of genetic variants with the risk of common obesity have been generated. Thus, the aim of this study was to evaluate the association between three *FTO* SNPs, including rs9939609 and rs1421085, prominent in the literature, and rs1861868, yet poorly studied, with the susceptibility to obesity in a sample of Portuguese children.

## Materials and Methods

### Study subjects

Children aging 6 to 12 years old were randomly selected from several public schools in the central region of Portugal. A total of 1433 Portuguese children of European descent comprising 747 girls and 686 boys were recruited [18], and were classified using age and sex specific BMI cut-offs provided by the International Obesity Task Force (IOTF) [20]. From the 1433 analysed children, three BMI groups were formed: 320 subjects were classified as overweight (resulting from the BMI in adult's cut-points between ≥25 kg/m^2^ and <30 kg/m^2^), 154 as obese (BMI ≥30 kg/m^2^), and 959 as normal weight (BMI <25 kg/m^2^).

The study protocol was approved by *Direção-Geral de Inovação e de Desenvolvimento Curricular*, the ethical Committee of the Portuguese Ministry of Education, and was conducted in accordance with the institutional guidelines of the University of Coimbra. Written informed consent was previously obtained from the children's parents.

### Anthropometric Measurements

Height (cm) and weight (kg) were taken with participants dressed in lightweight clothing without shoes. Waist circumference (cm) was measured midway between the lowest rib and the iliac crest, to the nearest 0.1 cm after inhalation and exhalation. Hip circumference (cm) was measured at the point over the buttocks yielding the maximum circumference. The BMI was calculated with the weight in kilograms divided by the square of height in meters (kg/m^2^). Abdominal obesity was defined using the sex and age-specific ≥90^th^ waist circumference percentile [21].

### Selected and genotyping of the FTO polymorphisms

Samples were analysed for three SNPs located within first intron of the *FTO* gene: two that have been closely associated with obesity and prominent in the literature, rs9939609 (position: chr16:53820527), described by Frayling et al. [5], and rs1421085 (position: chr16:53800954), reported in the work of Dina et al. [7], and the yet poorly studied rs1861868 polymorphism (position: chr16:53790402), described in two studies [8], [9].

A buccal swab sample was collected from each child for genetic studies. The genomic DNA was extracted from buccal cells using the PureLink Pro 96 Genomic DNA Kit (Invitrogen Corporation, Carlsbad, CA, USA), according to the instructions of the manufacturer, and was only used for the SNPs genotyping.

Samples were genotyped for the three *FTO* SNPs by allelic discrimination assays using TaqMan probes (C_30090620_10, C_11717119_10 and C_8917103_10; Applied Biosystems, Foster City, USA). Fluorescence was visualized through a MiniOpticon real time PCR system (Bio-Rad, Hercules, CA, USA).

To assess genotyping reproducibility, a selection of 10% random samples was re-genotyped for all SNPs with 100% concordance by the Single Strand Conformation Polymorphism (SSCP) method or sequencing by the Sanger's dideoxy chain termination reaction using Big-Dye Terminator v1.1 Cycle Sequencing kit (Applied Biosystems, Foster City, USA) and the ABI 310 sequencer (Applied Biosystems, Foster City, USA), using oligonucleotides 5′-CATCAGTTATGCATTTAGAATGTCTG-3′ (forward) and 5′-TCCCACTCCATTTCTGACTGT-3′ (reverse) for rs9939609, 5′-AATCTCATTGTTCCTCCTGCT-3′ (forward) 5′-ACAGTGGAGGTCAGCACAGA-3′ (reverse) for rs1421085, and 5′-CGCATCTCTGCAACTCTTTT-3′ (forward) and 5′-TGCTTTGTTAAGGCCATAGG-3′ (reverse) for rs1861868.

### Statistical analysis

The allele and haplotype frequencies were estimated by direct gene counting. The software package Arlequin, version 3.5 (http://cmpg.unibe.ch/software/arlequin35/) [22], was used to calculate allele frequencies, Hardy-Weinberg equilibrium probability values and *D*' and *r^2^* values for linkage disequilibrium (LD). Haplotype phase was determined by statistical inference via the ELB algorithm implemented in Arlequin, version 3.5. The quantitative variables were expressed as means and standard deviation, and qualitative variables were presented as absolute numbers and frequencies. The one-way analyses of variance (ANOVA), followed by *post hoc* Bonferroni test, was used to examine anthropometric traits for differences between genotype groups. For the analyses of covariance (age and gender) General Linear Models were used. Logistic regression models, adjusted for age and gender, were used to calculate *p* values, odds ratio (OR), and 95% confidence intervals (CI), for each SNP and haplotypes. All association analyses were performed using the Statistical Package for the Social Sciences (SPSS, for windows version 18.0). Statistical significance was taken at *p*-values ≤0.05 for all comparisons.

## Results

The analysed children were divided into three groups according to the definition of BMI specified by IOTF cut-offs [20]. From a total of 1433 children measured for anthropometric traits, genotyping was performed in a total of 730 children comprising 320 subjects classified as overweight (≥25 kg/m^2^ BMI <30 kg/m^2^), 154 classified as obese (BMI ≥30 kg/m^2^) and a control group of 256 subjects randomly selected from the total normal weight children (*N* = 959, BMI <25 kg/m^2^). A descriptive study of the total genotyped sample, stratified by phenotype distribution, is shown in Table 1. The genotyping success rate of the three selected SNPs varied between 93.3% and 99.6%. Genotype frequencies for the total sampled population were in accordance with Hardy-Weinberg equilibrium (*p* = 1.000 for rs9939609, *p* = 0.598 for rs1421085, and *p* = 0.937 for rs1861868). The minor allele frequency observed for the three SNPs in the total sample was 44.8% for the rs9939609-A allele, 45.4% for the rs1421085-C allele, and 46.1% for the rs1861868-G allele.

**Table 1:** General characteristic of the sampled children by phenotype distribution.

Characteristics	Overall	Phenotype distribution*		
		Normal	Overweight	Obese
*N*	730	256	320	154
Girls (%)	50.7	55.9	45.3	53.2
Age (years)	9.1±1.7	8.6±1.6	9.5±1.6	9.0±1.7
Height (cm)	136.2±11.7	131.1±11.1	139.5±11.1	137.9±10.6
Weight (kg)	37.2±11.3	28.1±6.6	40.2±9.3	46.1±11.0
BMI (kg/m^2^)	19.6±3.4	16.1±1.5	20.3±1.8	23.8±2.5
BMI Z-score	0.93±0.97	−0.15±0.78	1.3±0.23	1.99±0.23
Waist circumference (cm)	67.2±7.8	60.3±4.5	68.9±5.4	75.1±6.6
Hip circumference (cm)	79.0±10.3	70.4±6.5	81.9±8.0	87.1±9.4
WHR	0.85±0.06	0.86±0.06	0.85±0.06	0.87±0.05

Data are presented as mean ± standard deviation.

* Phenotype distribution was determined using age and gender specific BMI cut-offs provided by the International Obesity Task Force (IOTF).

Abbreviations: BMI, body mass index; BMI Z-score, body mass index standard deviation score; WHR, waist-to-hip ratio.

We analysed anthropometric traits among different genotypes of *FTO* SNPs and found statistical significant differences in the mean score for rs9939609 and rs1421085 SNPs for increasing weight, BMI, BMI Z-score, waist circumference (WC) and hip circumference (HC) (*p*≤0.05 for all traits) (Table 2). The strongest associations were found with BMI (*p* = 0.005) and WC (*p* = 0.005), even after age and gender adjustment (Table 2). The rs9939609 per-A allele increases was ∼0.6 kg/m^2^ in BMI, ∼1.2 cm in WC and ∼1.7 kg in weight; similar values were obtained for each rs1421085-C allele: 0.55 kg/m^2^, 1.25 cm and 1.55 kg, for BMI, WC and weight, respectively. The rs1861868 SNP also showed associations with weight, BMI, WC and HC. Non-significant results were obtained after adjusting for age and gender (*p*≥0.05 for all traits), nevertheless for weight and BMI marginally significant results (*p* = 0.081 and *p* = 0.096, respectively) were obtained (Table 2).

**Table 2:** Comparison of anthropometric parameters among different genotypes of the *FTO* rs9939609, rs1421085 and rs1861868 polymorphisms in the sampled Portuguese children.

	rs9939609					rs1421085					rs1861868				
Characteristics	TT	AT	AA	*P*	*P* ^1^	TT	CT	CC	*P*	*P* ^1^	GG	AG	AA	*P*	*P* ^1^
*N* (%)	220 (30.5)	357 (49.5)	144 (20.0)			213 (29.3)	368 (50.6)	146 (20.1)			140 (21.0)	336 (50.3)	192 (28.7)		
Height (cm)	134.7± 12.2	136.8± 11.5	137.6± 11.0	**0.031**	**0.044**	134.8± 12.1	136.5± 11.7	137.4± 10.6	0.090	0.077	134.3± 11.3	136.3± 11.9	137.4± 11.0	0.051	0.125
Weight (kg)	35.5± 11.7	37.8± 11.1	38.9± 11.1	**0.010**	**0.019**	35.6± 11.8	37.4± 11.2	38.7± 10.9	**0.031**	**0.032**	34.8± 10.3	37.5± 11.7	38.7± 11.3	**0.007**	0.081
BMI (kg/m^2^)	19.0± 3.4	19.7± 3.4	20.2± 3.5	**0.005**	**0.018**	19.1± 3.5	19.6± 3.4	20.2± 3.5	**0.010**	**0.022**	18.9± 3.2	19.7± 3.5	20.0± 3.5	**0.007**	0.096
BMI Z-score	0.77± 1.05	0.98± 0.93	1.09± 0.91	**0.006**	**0.011**	0.76± 1.1	0.96± 0.91	1.09± 0.9	**0.005**	**0.009**	0.76± 1.1	0.96± 0.98	1.02± 0.88	0.053	0.133
WC (cm)	66.0 ± 7.9	67.7 ± 7.6	68.4 ± 7.7	**0.005**	**0.016**	66.0± 7.9	67.5± 7.6	68.5± 7.8	**0.007**	**0.013**	65.7± 7.4	67.4± 7.9	68.1± 7.7	**0.020**	0.214
HC (cm)	77.4± 10.5	79.6± 10.0	80.5± 9.0	**0.008**	**0.017**	77.6± 10.6	79.3± 10.1	80.4± 9.8	**0.030**	**0.035**	76.9± 9.7	79.3± 10.3	80.3± 10.5	**0.009**	0.108
WHR	0.86± 0.05	0.85± 0.06	0.85± 0.05	0.896	0.966	0.85± 0.05	0.85± 0.06	0.86± 0.05	0.986	0.799	0.86± 0.05	0.85± 0.05	0.85± 0.06	0.667	0.759

Abbreviations: BMI, body mass index; BMI Z-score, body mass index standard deviation score; WC, waist circumference; HC, hip circumference; WHR, waist-to-hip ratio.

*p*-values were analyzed by one-way ANOVA.

*p* ^1^–values were adjusted for age and gender. *p*-value significant (*p*<0.05) in bold.

We performed association analysis under the additive model using BMI case-control groups (Table 3). When compared obese *vs*. normal-weight groups the rs9939609 AA genotype showed significant association with risk of obesity (OR 1.97; 95% CI, 1.08–3.59; *p* = 0.026), but not the AT genotype (OR 1.47; 95% CI, 0.91–2.38; *p* = 0.116). Accordingly, 23.4% of the obese individuals were AA homozygotes compared to 16.2% of the control subjects. Association was also observed with increased risk of being obese for the rs1421085 CC genotype (OR 2.11; 95% CI, 1.17–3.81; *p* = 0.013), but not for the CT genotype (OR 1.48; 95% CI, 0.90–2.43; *p* = 0.123). For this SNP, 26.6% of the obese individuals had the CC genotype, against 17.7% with normal weight. For the rs1861868 SNP, it was not found a significant association with obesity (OR 1.35; 95% CI, 0.74–2.47; *p* = 0.332) (Table 3). Association analysis under an allelic model, comparing obese *vs*. normal-weight groups, showed similar significant results for rs9939609 (OR 1.44; 95% CI, 1.08–1.91; *p* = 0.012) and rs1421085 (OR 1.48; 95% CI, 1.12–1.98; *p* = 0.007), but not for rs1861868 (OR 1.20; 95% CI, 0.89–1.61; *p* = 0.228). We detected no significant association when comparing overweight *vs*. normal-weight groups (*p*≥0.05) (Table 3).

**Table 3:** Allelic and genotypic frequencies for the *FTO* rs9939609, rs1421085 and rs1861868 polymorphisms, and odd ratio (OR) values among phenotypic groups.

SNPs		Phenotype distribution*				Phenotype distribution*			OR (95% CI)	
	Allele	Normal	Overweight	Obese	Genotype	Normal	Overweight	Obese	Obese *vs*. normal	Overweight *vs*. normal
rs9939609	A	0.409	0.453	0.497	AA	40 (16.2)	68 (21.3)	36 (23.4)	1.97 (1.08–3.59) *p* = **0.026**	1.36 (0.82–2.27) *p* = 0.230
*N* = 721					TA	122 (49.4)	154 (48.1)	81 (52.6)		
	T	0.591	0.547	0.503	TT	85 (34.4)	98 (30.6)	37 (24.0)		
rs1421085	C	0.339	0.342	0.373	CC	45 (17.7)	60 (18.8)	41 (26.6)	2.11 (1.17–3.81) *p* = **0.013**	1.02 (0.61–1.69) *p* = 0.939
*N* = 727					TC	127 (50.0)	161 (50.5)	80 (51.9)		
	T	0.661	0.658	0.627	TT	82 (32.3)	98 (30.7)	33 (21.4)		
rs1861868	A	0.506	0.558	0.552	AA	60 (26.0)	89 (30.3)	43 (30.1)	1.35 (0.74–2.47) *p* = 0.332	1.29 (0.77–2.17) *p* = 0.328
*N* = 668					GA	114 (49.4)	150 (51.0)	72 (50.3)		
	G	0.494	0.442	0.448	GG	57 (24.7)	55 (18.7)	28 (19.6)		

Data for allele frequencies are in percentage and genotype frequencies are number of subjects, divided into genotype groups (% in each phenotype distribution).

* Phenotype distribution was determined using age and gender specific BMI cut-offs provided by the International Obesity Task Force (IOTF).

Abbreviations: SNPs, polymorphisms; OR, odds ratio; CI, confidence interval.

Logistic regression was used to compare genotype distribution.

*p*-values were under the additive model, and adjusted for age and gender. *p*-value significant (*p*<0.05) in bold.

Haplotype analysis associating the three studied *FTO* SNPs (rs1861868-rs1421085-rs9939609), revealed all the eight possible haplotypes, being the most commons GTT (33%), ACA (32%), ATT (19%) and GCA (12%) (Table 4). Three haplotypes had an estimated frequency below 1% (GCT, ATA and GTA). Compared with the most common and non-risk haplotype (GTT), two haplotypes (ACA and GCA) were significantly associated with a higher risk of being obese (OR 1.534; 95% CI, 1.06–2.22; *p* = 0.023; OR 1.739; 95% CI, 1.06–2.87; *p* = 0.030, respectively).

**Table 4:** Haplotype frequencies associating *FTO* rs1861868-rs1421085-rs9939609 polymorphisms in the sampled Portuguese children.

Haplotype	Frequency	OR	95% CI	*p*-value
GTT	0.33	Reference		
ATT	0.19	1.133	0.73–1.75	0.572
ACA	0.32	1.534	1.06–2.22	**0.023**
GCA	0.12	1.739	1.06–2.87	**0.030**
ACT	0.02	2.000	0.68–5.89	0.209
Rare	0.02	1.200	0.42–3.42	0.733

Rare: haplotypes with a frequency under 1% (GCT, ATA and GTA).

Abbreviations: OR, odd ratio; CI, confidence interval.

*p*-value significant (*p*<0.05) in bold.

Regarding allelic combinations, SNPs rs9939609 (position: chr16:53820527) and rs1421085 (position: chr16:53800954), distant from one another about 19.6 kb, were found in high LD (*D*' = 0.91; *r^2^* = 0.82). The rs1861868 SNP (position: chr16:53790402) was found in low LD (*D*' = 0.39; *r^2^* = 0.11) with rs9939609, distant about 30.1 kb, as well with rs1421085 (*D*' = 0.44; *r^2^* = 0.13), distant about 10.6 kb.

## Discussion

Recently, the growth in studies regarding the association of obesity, or obesity-related traits, with SNPs in the *FTO* gene has been reported for several populations across the world [5]–[17]. Most studies confirmed that *FTO* SNPs are strongly associated with BMI and/or obesity [5]–[17]. However, in Portugal there are no studies to confirm the association between genetic variants and common obesity, which could permit the comparison with data from other European populations. Despite the similar genetic background between European populations, it is known that for several polymorphisms, frequencies can vary within different Caucasian populations [23], [24]. Moreover, a few studies failed to associate some *FTO* polymorphisms and obesity [25], [26], highlighting the need of more studies in different populations to better understand the role of *FTO* gene in obesity. The present study is the first to test whether common *FTO* gene SNPs are associated with obesity or to related anthropometric traits in children of Portuguese origin.

Our research showed a significant genetic association of rs9939609 and rs1421085 SNPs, in strong linkage disequilibrium (*r^2^* = 0.82), with the risk of obesity in Portuguese children. Consistently, we also observed significant association with several anthropometric measurements including weight, BMI, WC and HC. These results are similar to those found in previous studies performed in other European populations reporting the association of *FTO* SNPs with obesity [5]–[7], [27]. In our study, the effect obtained for each copy of rs9939609-A allele was ∼0.6 kg/m^2^ in BMI, ∼1.2 cm in WC and ∼1.7 kg in weight, similar to the effect stated by Frayling et al. [5].

Regarding rs1861868 SNP, association with BMI was first described in a sample of Old Order Amish with low physical activity [8] and replicated in a sample of Spanish children [9]. However, in this last study, it was not found a significant association with BMI or obesity. Our study showed an association with weight, BMI, WC and HC, with marginally significant results for weight (*p* = 0.081) and BMI (*p* = 0.096) after adjusting for age and gender. This suggests that stronger statistically significant results could be obtained by increasing the sample size.

Our results show that in Portuguese children the rs9939609 and rs1421085 SNPs are in association with obesity, with no differences between girls and boys, and in line with previously reported studies in other European populations [5]–[10]. We found an OR of 1.44 for the rs9939609 SNP under an allelic model. This result appears similar to the effects reported by Frayling et al [5] in UK children (OR  = 1.35; 95% CI, 1.14–1.61), and Hinney et al. [28] in German children/adolescents (OR  = 1.57; 95% CI, 1.30–1.90). We also found an OR of 1.48 for the rs1421085 SNP similar to that reported by Dina et al. [7] in French children (OR  = 1.43; 95% CI, 1.25–1.64), and Meyre et al. [29] in German children (OR  = 1.50; 95% CI, 1.25–1.79). The rs9939609 was the most replicated SNP associated with obesity across the world, nevertheless, in our study the strongest association was obtained with the rs1421085 SNP (OR 2.11; 95% CI, 1.17–3.81; *p* = 0.013, additive model), similar to the result obtained by Price et al. [30] in a sample of Caucasian women when analysing both SNPs.

None of the three study SNPs showed evidence of association with overweight in the sample. This means that the *FTO* risk allele predominates in individuals with higher BMI; hence the association was detected in severe obesity rather than in overweight population, similarly to the results obtained by Liu et al. [14].

The *FTO* risk allele frequencies observed in our study are within range of reported values in European populations [5]–[10]. Both rs9939609 and rs1421085 SNPs were found in high LD (*r^2^* = 0.82) in our study reflecting the high LD across the 19.6 kb region within the intron 1 of *FTO* gene. Polymorphisms rs9939609 and rs1421085 are both part of a set of BMI-associated SNPs within a 47 kb LD block encompassing parts of the first two introns as well as exon 2 of the *FTO* gene [31] suggesting that they all tag a same genetic signal in that region. The low LD (*r^2^* = 0.13) observed in our study between rs1861868 5′ apart 10.6 kb from rs1421085, complement the lower genetic predictive power of rs1861868 for the studied obesity related parameters, suggesting that LD block decline between these two SNPs. As we show (Table 4) the only two common haplotypes that seem to confer risk to obesity were ACA (*p* = 0.023) and GCA (*p* = 0.030), which include both risk alleles A and C for rs9939609 and rs1421085, respectively. For the haplotype ACT, presenting only one risk allele, no association (*p* = 0.209) with obesity was found. This seems to reflect that haplotypes combining the risk alleles for the two SNPs rs9939609 and rs1421085 have increased risk of obesity.

In 1962, Neel proposed the thrifty gene hypothesis [32] suggesting that populations whose ancestral environments were characterized by periods of feast and famine, experienced positive selection for thrifty alleles that promote the storage of fat and energy. Thus, under modern conditions, populations with such thrifty alleles are expected to have high rates of obesity. Regarding the ancestral alleles for SNPs rs9939609 and rs1421085 comparing sequence similarity with non-human primates, the ancestral rs9939609-A allele is associated with the obesity risk but not the ancestral rs1421085-T allele. This different genetic association pattern is not consistent with the thrifty gene hypothesis, as also suggested in a previous report [33], because under this hypothesis we should expect a similar pattern regarding ancestry of the risk alleles.

In conclusion, this is the first study reporting allele and genotype frequencies of the *FTO* polymorphisms in the Portuguese population. We found evidence that the previously reported common polymorphisms rs9939609 and rs1421085 in *FTO* gene increase the risk of obesity in the Portuguese children. Further studies on other polymorphisms from *FTO* and other genes are needed, to establish the genetic basis contributing to the risk of obesity in the Portuguese population.
